# Digital Therapeutic Intervention for Women in the UK Armed Forces Who Consume Alcohol at a Hazardous or Harmful Level: Protocol for a Randomized Controlled Trial

**DOI:** 10.2196/51531

**Published:** 2023-12-19

**Authors:** Grace Williamson, Ewan Carr, Nicola T Fear, Simon Dymond, Kate King, Amos Simms, Laura Goodwin, Dominic Murphy, Daniel Leightley

**Affiliations:** 1 King's Centre for Military Health Research King's College London London United Kingdom; 2 Biostatistics & Health Informatics King's College London London United Kingdom; 3 Academic Department of Military Health King's College London London United Kingdom; 4 School of Psychology Swansea University Swansea United Kingdom; 5 Department of Psychology Reykjavík University Reykjavík Iceland; 6 Academic Department of Military General Practice Defence Medical Services Birmingham United Kingdom; 7 Faculty of Health and Medicine Lancaster University Lancaster United Kingdom; 8 Combat Stress Leatherhead United Kingdom

**Keywords:** mental health, digital health, DrinksRation, women, woman, United Kingdom, Armed Forces, alcohol, randomized controlled trial, RCT, controlled trials, study protocol, alcohol misuse, smartphone app, mobile app, mobile health, mHealth, veterans, women veterans, mobile phone, digital therapeutic

## Abstract

**Background:**

Alcohol misuse is common in the United Kingdom Armed Forces (UKAF), with prevalence significantly higher than in the general population. To date, digital health initiatives to support alcohol misuse have focused on male individuals, who represent approximately 89% of the UKAF. However, female veterans drink disproportionally more than female members of the public.

**Objective:**

This 2-arm participant-blinded (single-blinded) confirmatory randomized controlled trial (RCT) aims to assess the efficacy of a brief alcohol intervention (DrinksRation) in reducing weekly self-reported alcohol consumption between baseline and a 3-month follow-up (day 84) among women who have served in the UKAF.

**Methods:**

In this 2-arm single-blinded RCT, a smartphone app that includes interactive user-focused features tailored toward the needs of female veterans and designed to enhance participants’ motivations to reduce the amount of alcohol they consume is compared with the UK Chief Medical Officer guidance on alcohol consumption. The trial will be conducted among women who have served at least 1 day of paid service in the UKAF. Recruitment, consent, and data collection will be carried out automatically through the DrinksRation app or the BeAlcoholSmart platform. The primary outcome is change in self-reported weekly alcohol consumption between baseline (day 0) and the 3-month follow-up (day 84) measured using the Timeline Follow Back for alcohol consumption. The secondary outcome is the change in the Alcohol Use Disorders Identification Test score measured at baseline and 3-month follow-up between the control and intervention groups. The process evaluation measures include (1) app use and (2) usability ratings as measured by the mHealth App Usability Questionnaire.

**Results:**

RCT recruitment will begin in January 2024 and last for 5 months. We aim to complete all data collection, including interviews, by May 2024.

**Conclusions:**

This study will assess whether a smartphone app tailored to the needs of women who have served in the UKAF is efficacious in reducing self-reported alcohol consumption. If successful, the digital therapeutics platform could be used not only to support women who have served in the UKAF but also for other conditions and disorders.

**Trial Registration:**

ClinicalTrials.gov NCT05970484; https://www.clinicaltrials.gov/study/NCT05970484

**International Registered Report Identifier (IRRID):**

PRR1-10.2196/51531

## Introduction

### Background

There are 2.5 million military veterans in the United Kingdom (defined by the British government as those who have served in the military for at least 1 paid day), of whom 11% are estimated to identify as female [1]. Female veterans have served in the United Kingdom Armed Forces (UKAF) for >100 years. Although their valuable contribution has been recognized by successive governments and improved military culture. However, with evolving service requirements, they have had a significant impact on the health and well-being of many female veterans [2,3]. Nevertheless, there is a dearth of evidence on the impact of alcohol use on female veterans’ health.

The limited existing evidence suggests that female veterans’ alcohol use is increasing and that they are significantly more likely to report symptoms of hazardous drinking than female civilians [4]. Increased rates of hazardous drinking were also observed by Palmer et al [5], who found that half of the female veterans (389/779, 49.9%) were misusing alcohol at a hazardous or higher level, which is considered harmful to their health [5]. To place this into context, the UK Chief Medical Officer recommends that people do not regularly drink >14 units per week to keep health risks from drinking alcohol to a low level.

Alcohol misuse often co-occurs with common mental health disorders, including posttraumatic stress disorder (PTSD), anxiety, or depression, and alcohol is frequently used as a coping mechanism [2]. Common mental health disorders are more common in female than male veterans [4]. Research has also shown that female veterans face barriers to accessing mental health support, often owing to misusing alcohol [6,7]. Ultimately, although female veterans drink less than male veterans, their rates of hazardous drinking are higher than those of the general population, putting them at increased risk of poorer health.

The impact of alcohol misuse among female veterans on the wider society (eg, health care use, productivity, and welfare) is unknown. In England, heavy drinking (deemed as drinking >14 units of alcohol/wk) is estimated to cost the National Health Service (NHS) £3.5 billion per year (US $3.9 billion; 3.6% of its annual budget) [8] and is more common in people with mental health difficulties [9]. As female veterans drink more than their civilian counterparts, the relative costs are likely to be even higher. Innovative solutions are urgently required.

In recent years, there has been a growing treatment gap in the United Kingdom, with patients waiting longer for treatment and support for alcohol misuse. To overcome this gap, we developed the DrinksRation platform [10], an automated brief digital intervention designed to support help-seeking veterans in managing and reducing the amount of alcohol they drink [11-14]. DrinksRation is unique in that the app content is tailored using behavior change techniques (BCTs) to promote positive changes in behavior. DrinksRation is the only app targeting alcohol misuse in the UKAF community. It is designed to (1) overcome geographic limitations, (2) use wearable technology (eg, Fitbit and Apple Watch) to inform decision-making and personalization, (3) avoid the stigma associated with receiving help in person, and (4) provide convenience because users can use the app as they prefer (discreetly or openly). The app is freely available on the Apple App Store and Google Play Store. The app has received support and endorsement from Combat Stress and is currently being trialed among serving personnel [15]. DrinksRation is supported by a robust evidence base, including a randomized controlled trial (RCT) that demonstrated that the app is efficacious in reducing alcohol consumption [12].

The DrinksRation app was developed to support veterans who have sought help for a mental health problem and was not designed with potential gender differences in mind. A recent viewpoint highlighted a critical need for feminist intersectionality in digital health to incorporate the unique needs of female individuals [16]. Digital health technologies can bolster gender equality through providing increased access to health care, empowering decision-making about one’s health data, overcoming the specific barriers facing female veterans, and reducing the burden on health care systems [17-19].

### Objectives

This project aims to tailor the DrinksRation app to reflect the specific needs of female veterans and evaluate these changes using a confirmatory RCT. It is hypothesized that a refined version of DrinksRation will be efficacious at reducing self-reported weekly alcohol consumption between baseline and the 3-month follow-up (day 84) among female veterans who drink at a hazardous or harmful level.

## Methods

### Ethical Considerations

**Figure 1:**
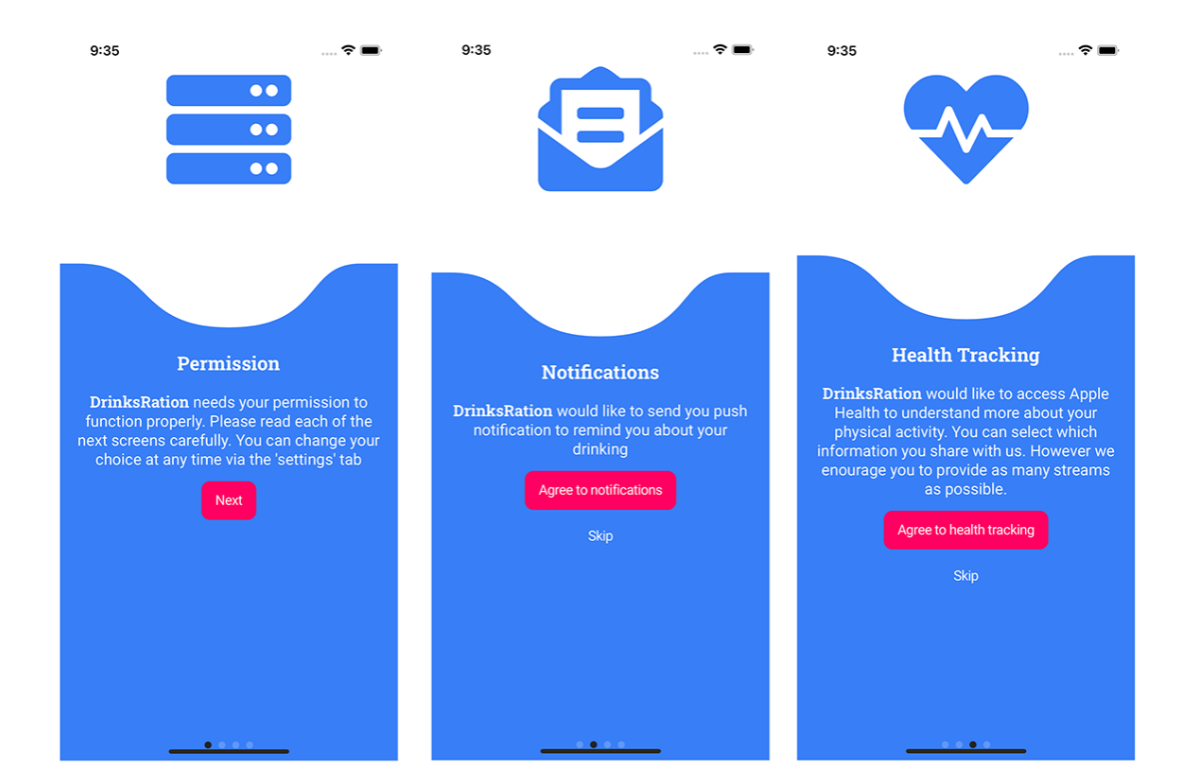
Consent flow screenshot examples presented to all study participants.

This study was approved by the ethics committee of King’s College London (LRS/DP-22/23-36879). The study has been registered prospectively on ClinicalTrials.gov (NCT05970484). After completing outcome questionnaires (day 84) at the primary end point (day 84), participants will receive a US $25 (£20 GBP) Love2Shop voucher. There will be no cash alternative offered to participants. We will seek informed consent from all participants via the eligibility and consent survey before the collection of any personal data. For participants in the intervention arm, we will additionally ask for specific on-device permissions to enable the full functionality of the DrinksRation app (eg, to receive push notifications or share health tracking data; refer to the example screenshots presented in Figure 1). Participants can change these optional permissions at any time via the settings page of the app.

Participants will be informed via the participant information sheet that they can withdraw from the study or withdraw from data collection at any time. For participants who withdraw from the study, we will (1) attempt to collect follow-up information if they agree to continued data collection and (2) retain their existing data if they agree to their use. Alternatively, withdrawn participants can delete their study data by contacting the research team. Participants in the control arm will need to withdraw by contacting the research team, whereas those in the intervention arm can withdraw via the app.

If participants in the intervention arm withdraw, they will be asked to delete the intervention app and denied further access. All data, including those from withdrawn participants, except for those who make a request for their data to be deleted, will be included in the final analysis.

### Study Design

#### Overview

This is a 2-arm participant-blinded (single-blinded) confirmatory RCT comparing the DrinksRation smartphone app (intervention arm) with a progressive web application (PWA) presenting NHS-focused drinking advice (named *BeAlcoholSmart*; control arm). The DrinksRation app provides individualized normative advice with features designed to enhance participant motivation, interactive feedback, self-efficacy in modifying their alcohol consumption, and personalized gender-specific messaging. We hypothesize that the intervention arm will be efficacious in reducing alcohol consumption compared with the control arm.

Both the control and intervention arms will be delivered digitally. Participants in the control arm will be given access only to a PWA that comprises a calculator for calculating alcohol units consumed over the previous week, generic public health guidance [20], and weekly reminders to use the calculator (refer to the *Control Arm* subsection for details). Participants in the intervention arm will be given access to the full enhanced DrinksRation app, which includes a range of theoretically driven features and behavioral change messaging (discussed further in the *Intervention Arm* subsection). We have designed this study so that the control arm resembles the intervention arm (eg, an app delivered digitally and usable on a smartphone) but lacks the active ingredients present in the DrinksRation intervention arm (eg, a drinks diary, interactive drinking feedback, and goal setting). This approach was selected to ensure consistency between the arms (both groups receive a digital intervention) and to facilitate participant blinding to treatment assignment.

**Figure 2:**
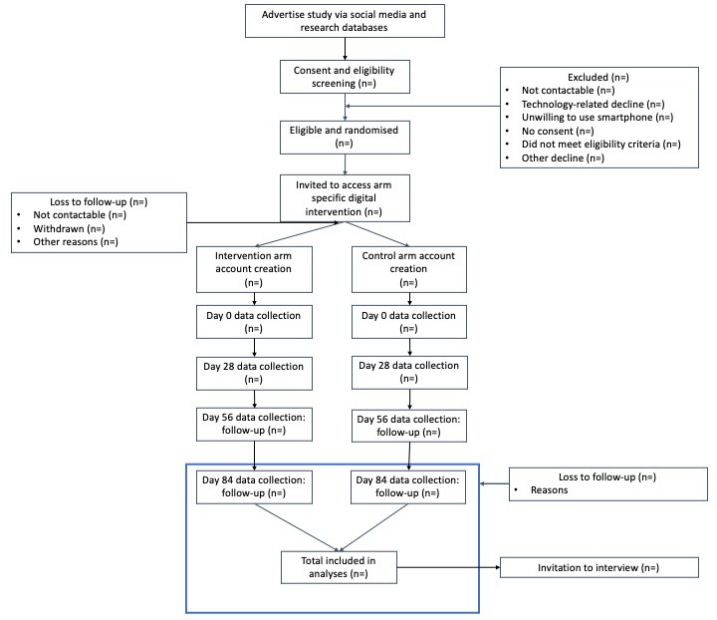
Study flow diagram. Primary outcome is assessed at day 84.

Participants in both arms will be asked to use the digital interventions for 12 weeks (the intervention period; 84 days). Figure 2 presents the study flow and data collection points.

#### Intervention Arm

The DrinksRation (formerly called *InDEx* [13,14,21]) app was developed following the Medical Research Council’s guidelines for developing and evaluating complex interventions and using a co-design methodology. It was developed by the King’s Centre for Military Health Research (King’s College London) and Lancaster University with support from experts in smartphone app development, epidemiology, addiction psychiatry, and military mental health. The app is designed to support veterans drinking at a hazardous or harmful level by providing bespoke advice and support.

**Figure 3:**
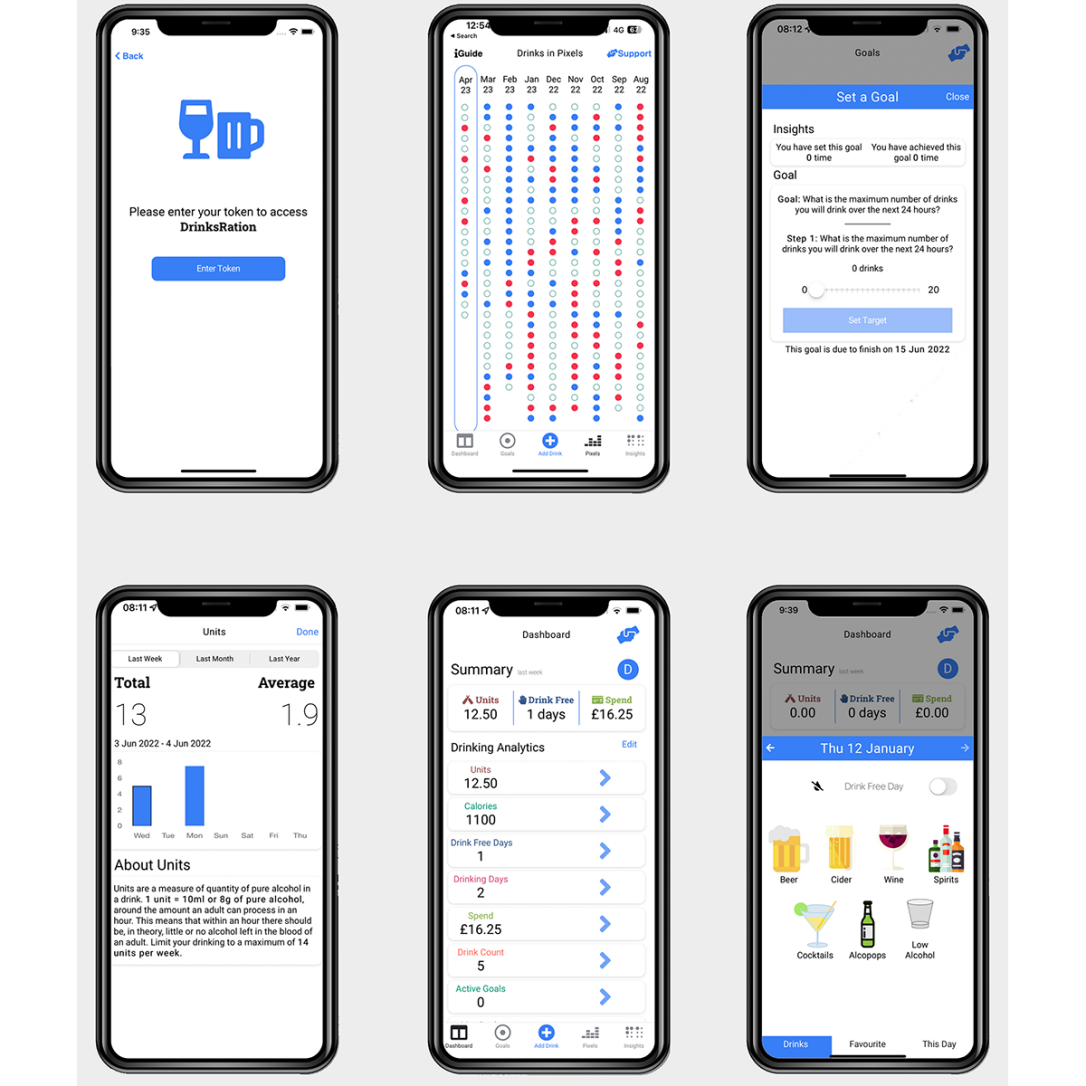
Example screenshots of the DrinksRation app.

The app seeks to enhance participants’ motivation and self-efficacy in modifying their alcohol consumption using BCTs presented via the in-app content (Multimedia Appendix 1) and through messages sent to participants. The iterative development process, theoretical framework, feasibility trial, pilot, and RCT are reported elsewhere [11-14,21]. Briefly, DrinksRation was developed and tested with 5 core modules (Textbox 1; refer to Figure 3 for example screenshots).

The app is compatible with all modern iOS- and Android-supported devices. Participants in the intervention arm will complete all questionnaires via the app, including additional questionnaires on their mood and general mental health, which are used to personalize the app content and push notifications. Participants will receive push notifications every few hours on the day that each questionnaire is due to prompt the completion of unanswered questions.

**Core modules of the DrinksRation app.**

**Account management**

- Participants can modify personal information (eg, first name and mobile phone number) and app parameters (eg, automatic log out, data-sharing permission, and withdrawing from the study).

**Questionnaire and individualized normative feedback**

- This feature captures the participant’s responses to a set of questions and aggregates the responses to produce personalized feedback on drinking behaviors.

**Self-monitoring and feedback**

- This feature records alcohol consumption by participants and provides a range of visual illustrations (eg, charts, figures, and text) to allow for the monitoring of consumption. Furthermore, the participant can select visual metrics relevant to their interest (eg, calories, cost, and exercise required).

**Goals (setting and review)**

- Participants can set goal or goals based on the implementation intentions (if and then) [22] methodology; visual feedback provides feedback on progress toward achieving the goal or goals set.

**Personalized messaging**

- Participants are sent tailored messages via push notification, which provide prompts to use the drinks diary, suggest alternative behaviors, and provide feedback on goals.

#### Control Arm

The control arm will receive access to the BeAlcoholSmart PWA. A PWA is a website that looks and behaves like a smartphone app. The PWA can take advantage of smartphone features such as push notifications and haptics without requiring the participant to download it via an app store. The BeAlcoholSmart PWA will be accessible to participants in the control arm via a unique link. The app will contain a 7-day alcohol unit calculator and generic public health guidance on safe drinking [20]. Participants will also receive reminders via email prompting them to consult the guidance provided as part of the BeAlcoholSmart PWA. Control participants will be invited to complete all questionnaires via Qualtrics (Qualtrics International Inc), with an email reminder being sent when they are due.

### Participant Co-Design

This study will evaluate an adapted version of the DrinksRation app that caters to the specific needs of female veterans. To ensure that any adaptations and new features are suitable for the target population, we will undertake participatory research—following the guidelines provided in the study by Bell and Pahl [23]—in the form of co-design and cocreation via a patient advisory board (PAB) and a stakeholder advisory board (SAB). The PAB will include those with lived experience of military service, whereas the SAB will include stakeholders and those representing the target population, such as charities treating alcohol misuse. PAB and SAB members will be recruited via our existing networks and communication channels.

We aim to recruit up to 5 members on both the PAB and SAB. Before undertaking any data collection, we will hold 2 roundtable discussions to collect input on the (1) development of app content, (2) refinement of existing app content, (3) development of new app features, and (4) suitability of tailored messaging.

In addition to PAB and SAB discussions, we will also ask members of the PAB to provide iterative feedback on updates to the DrinksRation app, study protocol, and study findings. Furthermore, we will hold a PAB and SAB meeting every quarter to provide updates on the study progress and to promote research awareness. PAB members will receive a US $13 (£10 GBP) digital Love2Shop voucher for each video session they attend. It is important to note that PAB members will not be allowed to participate in the RCT.

### Eligibility Criteria

The eligibility criteria are provided in Textbox 2.

**Inclusion and exclusion criteria.**

**Inclusion criteria**

- Aged ≥18 years
- Identify as female (self-reported sex)
- Live in the United Kingdom
- Consume ≥15 UK units (approximately 150 g) of alcohol/wk as measured using Timeline Follow Back for alcohol consumption [24] at baseline (day 0; the choice of a minimum threshold of 15 UK units of alcohol/wk reflects scientific research and UK national guidelines that indicate that regular drinking of >15 units/wk carries increased health risks [25])
- Are veterans of the United Kingdom Armed Forces (UKAF), defined as per UK definitions as having completed at least 1 day of paid employment in the UKAF (verified by self-report at eligibility screening)
- Have downloaded the mobile app onto an iOS or Android smartphone

**Exclusion criterion**

- Do not own a smartphone

### Sample Size Calculation

A power calculation was performed based on previously reported data from the DrinksRation RCT [12]. With 148 participants (n=74, 50% per arm), we will have at least 80% power to detect a difference in alcohol consumption of 3 units (approximately 30 g of alcohol/wk) between the intervention and control arms at the 84-day follow-up assessment. This calculation assumes an SD of 5.6 units, an attrition rate of 40% by day 84, a correlation between successive follow-up assessments of 0.7, and an α of 5%.

We have chosen a minimum difference of 3 units based on treatment effects observed in similar studies [26-28] and in reductions observed in the DrinksRation feasibility trial [11] and the confirmatory RCT among help-seeking veterans [12], which found reductions at 28 days of 22 units between the arms. We have chosen a smaller minimum difference of 3 units, recognizing that treatment effects for brief interventions reduce over time and that the target population for this study is not a help-seeking population.

### Recruitment

Participants will be recruited via an existing research cohort [26] and social media [29] following published recruitment strategies [29,30]. The research cohort will comprise participants from the King’s Centre for Military Health Research health and well-being longitudinal cohort study [26], and participants will be sent an email inviting them to take part. We will also seek to recruit participants via paid promotional advertisements on social media (eg, Twitter, Facebook, and Instagram) with a link enabling potential participants to complete the eligibility and consent survey.

Potential participants who respond to the email invitation or social media advertisements will be sent an email containing an explanation of the study, a link to the participant information sheet, and an invitation to complete the eligibility and consent survey (Multimedia Appendix 2). Eligible participants will then be randomized (refer to the Randomization and Blinding subsection) and sent instructions on accessing the BeAlcoholSmart or DrinksRation app using a unique QR code. Once they have accessed the platform, they can register an account and complete the baseline questionnaire (day 0).

### Randomization and Blinding

Randomization will be conducted automatically when a token code is generated alongside a unique proxy identifier (managed by researcher GW). At this point, participants are automatically randomized to receive the control or intervention arm and are blinded. The randomization procedure is carried out automatically via a randomization algorithm with no human involvement except to provide a proxy identifier. As allocations are generated dynamically, it is not possible to view future allocations. Researchers DL and GW will be unblinded to treatment allocation to enable the management of the BeAlcoholSmart and DrinksRation platforms as well as participant recruitment and retention, whereas researcher EC will be unblinded to treatment allocation to prepare statistical reports. Researchers DL and EC will not be involved in any participant engagement or recruitment. All other members of the study team will be blinded to treatment allocation.

### Messaging: Push Notifications and Email Messaging

Participants in the intervention arm (DrinksRation) will receive personalized push notifications to prompt them to use the drinks diary, engage with the app, and complete outcome measures, as well as to suggest alternative behaviors, provide feedback on goals, and promote a healthy lifestyle. We will use a bank of 180 messages developed previously [13], informed by the Health Action Process Approach framework and targeting specific BCTs.

DrinksRation uses baseline and weekly questionnaires to inform participant messaging and provide an individualized participant-centric approach. Baseline measurements are used to identify suitable messages, and as a participant engages with DrinksRation, regular measurements, including questionnaires (baseline and weekly questionnaires) and the drinks diary, are used to reflect current behavior and attitude. The notifications cover a wide range of topics to target beliefs and motivations with the primary aim of increasing the participant’s awareness of their drinking habits and behaviors. The notifications are divided into 3 categories: tailored (personalized to drinking habits as well as baseline and weekly questionnaires), tailored and triggered (tailored to baseline and contiguous measurements and a specific event occurring), and targeted (generic; sent on specific days to highlight inactivity, to remind participants to complete a questionnaire, or to alert participants to a new feature).

The system automatically decides when a message should be sent.

For the control arm (BeAlcoholSmart), participants will only receive notifications (via email) when they join the platform and when outcome measures are due. The welcome email will contain information on how to use the BeAlcoholSmart platform and when future notifications will be sent. The questionnaire emails will include a reminder to consult the BeAlcoholSmart platform and complete a questionnaire. No other notifications will be sent.

### Study Instruments

#### Overview

A summary of measures and data collection time points in this study is presented in Table 1.

**Table 1:** Summary of measures and data collection time points (days 0, 7, 14, 21, 28, 56 and 84).

Measure (Multimedia Appendix 3)			Days after randomization																																
			0					7					14					21					28					56					84 (primary end point)		
			I^a^		C^b^		I			C		I			C		I			C		I			C		I			C		I			C
**Questionnaires**																																			
	Informed consent or consents	✓		✓																															
	Sociodemographics	✓		✓																															
	Depression (PHQ-2^c^) [31]	✓		✓		✓					✓					✓					✓					✓			✓		✓			✓	
	Anxiety (GAD-2^d^) [32]	✓		✓		✓					✓					✓					✓					✓			✓		✓			✓	
	ITQ-PTSD^e^ [33]	✓		✓																	✓			✓		✓			✓		✓			✓	
	Readiness-to-Change Ruler [34]	✓		✓																	✓			✓		✓			✓		✓			✓	
	Self-Efficacy Ruler [34]	✓		✓																	✓			✓		✓			✓		✓				
	AUDIT^f^ [35]	✓		✓																	✓			✓		✓			✓		✓				
	EQ-5D-5L [36]	✓		✓																	✓			✓		✓			✓		✓			✓	
	7-d TLFB^g^ for alcohol consumption [24]	✓		✓																	✓			✓		✓			✓		✓			✓	
	Adverse event reporting																				✓			✓		✓			✓		✓			✓	
	Usability evaluation																																		
	Qualitative interviews^h^																														✓			✓	
	MAUQ^i^ [37]																				✓			✓		✓			✓		✓			✓	
**Remote data collection (continuous)**																																			
	Wearable sensors^j^	✓		✓		✓			✓		✓			✓		✓			✓		✓			✓		✓			✓		✓			✓	
	Smartphone sensors^j^	✓		✓		✓			✓		✓			✓		✓			✓		✓			✓		✓			✓		✓			✓	

^a^I: intervention arm data collected via app.

^b^C: control arm data collected via Qualtrics (Qualtrics International Inc) through BeAlcoholSmart platform.

^c^PHQ-2: Patient Health Questionnaire-2.

^d^GAD-2: Generalized Anxiety Disorder-2.

^e^ITQ-PTSD: International Trauma Questionnaire for Posttraumatic Stress Disorder.

^f^AUDIT: Alcohol Use Disorders Identification Test.

^g^TLFB: Timeline Follow Back.

^h^A total of 10 participants will be invited to participate in a qualitative interview after day 84.

^i^MAUQ: mHealth App Usability Questionnaire.

^j^Additional participant consent is required.

#### Baseline Measures

After they have downloaded the respective platforms, participants will be asked to complete a baseline questionnaire to assess physical and mental health, readiness to change, self-efficacy, health status, and sociodemographics (eg, age, gender, ethnicity, employment status, and occupation). The questionnaire set is available in Multimedia Appendix 3, and the schedule is presented in Table 1.

#### Outcome Measures

The primary outcome in this study is self-reported alcohol consumption measured by the 7-day Timeline Follow Back for alcohol consumption. At baseline (day 0) and 28, 56, and 84 days after randomization, participants will be asked to report the number and types of drinks that they consumed in each of the previous 7 days via DrinksRation or BeAlcoholSmart. Using standard unit measurements (Multimedia Appendix 4), we will calculate weekly alcohol consumption for baseline and each follow-up by summing the number of units assigned to each drink. To maximize the completion of the primary outcome at each follow-up, participants will first be asked to complete the primary outcome measure before completing the rest of the questionnaire. The secondary outcome is the change in the Alcohol Use Disorders Identification Test score, measured at baseline (day 0) and day 84 follow-up between the control and intervention groups.

#### Usability

We will examine the usability of the DrinksRation and BeAlcoholSmart platforms using the mHealth App Usability Questionnaire [37] at day 28. Questionnaire responses will be aggregated into (1) overall usability, (2) ease of use, (3) interface and satisfaction, and (4) usefulness. The results will be summarized with means and SDs.

### Qualitative Interviews

A random sample of 10 participants from the intervention arm will be invited to participate in a debrief session with the research assistant (RA; researcher GW) at the end of the intervention period. The 60-minute interview will address the participants’ experiences of using the DrinksRation app, their views on its acceptability and usability, and what further modifications could be made to improve their experiences of using the app. Interviews will be conducted on the web (via Microsoft Teams or Zoom [Zoom Video Communications, Inc]), and transcripts will be generated and used for analysis. A thematic analysis approach will be used. The findings from the qualitative interviews will be reported separately from the main trial results. Any participant who completes the day 84 outcome measure will receive a US $13 (£10 GBP) Love2Shop voucher as a gesture of goodwill.

### Adverse Events

It is not expected that participation in this study will lead to an increase in alcohol consumption or any untoward or unintended consequence. In this study, we have defined 4 categories of risk events (Textbox 3).

Adverse events and serious adverse events will be monitored throughout the intervention period via (1) direct contact between participants to the research team; and (2) a questionnaire presented to participants via the app on days 28, 54, and 84 after randomization. All reported events will be reviewed by a clinician (researcher DM), who, where required, will contact participants to perform a clinical interview and risk assessment. If concerns remain, a signposting booklet to relevant charities will be provided to the participants. The study RA will remove potentially unblinding information before sending it to the study clinician. The study clinician or the RA may register an adverse event based on the definitions outlined in Textbox 3.

**Categories of risk events.**

**Adverse event**

- Any untoward medical occurrence in a trial participant that does not necessarily have a causal relationship with this treatment (eg, consuming >25 units of alcohol in a 24-h period).

**Adverse reaction**

- Any untoward and unintended medical occurrence in a trial participant that is related to the intervention (eg, reaction to a questionnaire).

**Unexpected adverse reaction**

- An adverse reaction where the nature and severity of the reaction are inconsistent with the information known about the study.

**Serious adverse event, serious adverse reaction, or unexpected serious adverse reaction**

- Any adverse event, adverse reaction, or unexpected adverse medical event reaction that (1) results in death, (2) is life threatening or requires immediate safeguarding, (3) requires hospitalization or prolongs existing hospitalization, or (4) results in persistent or significant disability or incapacity.

### Project Management Group

Project oversight will be provided by a project management group (PMG). The PMG will be chaired by the principal investigator and attended by all coinvestigators. The PMG will meet every quarter and will be responsible for all aspects of project management, including (1) monitoring the delivery of the scientific results needed to progress the study; (2) monitoring the study against the schedule and act if required; (3) monitoring the study against agreed milestones and the identification of risks to achieving milestones as well as solutions for managing risks and issue resolution; (4) providing access to appropriate resources necessary for project progression, including internal and outsourced resources and the management of these resources; and (5) regular review of a blinded report from the study clinician about adverse and serious adverse events (ie, total numbers pooled across arms).

PMG members will be blinded to treatment allocation.

### Executive Steering Committee

An executive steering committee will oversee safety requirements and make recommendations to the PMG. This committee will consist of an independent chair and the study clinician. The committee will receive regular reports from the trial statistician (researcher EC) and the RA on risk and appraise study functions. Concerns and recommendations will be raised with the principal investigator and discussed at PMG meetings.

### Protocol Amendments

Protocol amendments will be discussed and approved by the PMG as well as the PAB and minuted. They will be reported in subsequent closed reports (eg, prepared by the unblinded trial statistician). Any approved amendments will be submitted to the King’s College London ethics committee for approval and published on the Open Science Framework.

### Statistical Analysis

We will describe participant characteristics using appropriate summary statistics (eg, frequency and percentage for categorical items and mean and SD or median and IQR for continuous items).

We will analyze the primary outcome using a linear mixed effect model. The outcome will be the 3 repeated outcome assessments (collected on days 28, 56, and 84). The model will include (1) the baseline measure of the outcome (day 0); (2) a dummy variable indicating treatment allocation (0=control arm and 1=intervention arm); (3) time, measured as weeks since baseline; (4) a treatment × time interaction; and (5) baseline characteristics associated with missingness. The model will additionally include a participant random intercept to account for repeated assessments clustered within individual participants and an unstructured residual covariance matrix. The mixed model will use all available information, retaining participants with at least 1 follow-up assessment, to improve efficiency. Treatment effects (adjusted between-group mean differences in alcohol units) will be estimated from the model for each follow-up separately. Cohen *d* effect sizes at 28, 56, and 84 days will be calculated as the adjusted mean difference divided by the sample SD of the outcome at baseline. The threshold for statistical significance is *P*<.05.

The intention-to-treat analyses will include all participants who complete at least 1 follow-up assessment (day 28, day 56, or day 84).

Analyses will be conducted using Stata (StataCorp LLC) or R statistical software (R Foundation for Statistical Computing).

### Process Evaluation

We will examine process evaluation measures as a proxy for app use. These will be reported in three categories: (1) app use based on app analytics data provided by Google Analytics, (2) drinking analytics based on server interactions, and (3) notifications sent by the server. Where appropriate, these will be summarized as either median with IQR or mean with SD.

### Access to Study Data

All study data will be made available in an anonymized format alongside any source code via the Open Science Framework. There are no contractual agreements that limit access to, or the sharing of, data.

### Data Management

Data collected in this study will be stored on Google Firebase servers located in the United Kingdom, with audited and copied in real time to Google BigQuery data storage to ensure data integrity. Access to these systems is protected using Google authentication and a zero-trust modal. Upon completion of the study, data will be extracted from the Google Firebase servers and Google BigQuery and compared for assurance and data quality.

During active data collection, the research team (DL and GW) will monitor incoming data to ensure that each platform functions correctly. The research team will not contact participants outside of automatic messaging unless they reach out to the research team with queries (managed by GW; eg, with technical issues concerning how to perform a task or if risk alerts are triggered).

## Results

RCT recruitment will begin in January 2024 and last for 5 months. We aim to complete all data collection, including interviews, by May 2024. The results of this study will be communicated via publication, lay articles disseminated via stakeholders and collaborators, and a participant newsletter.

The study will be reported following the TIDieR (Template for Intervention Description and Replication) [38] checklist as well as the CONSORT (Consolidated Standards of Reporting Trials) [39] and CONSORT-EHEALTH (Consolidated Standards of Reporting Trials of Electronic and Mobile Health Applications and Online Telehealth) [40] checklists.

## Discussion

### Summary

This project aims to tailor the DrinksRation app to reflect the specific needs of female veterans and evaluate these changes using a confirmatory RCT. We hypothesize that a refined version of DrinksRation will be efficacious at reducing self-reported weekly alcohol consumption among female veterans who drink at a hazardous or harmful level. Alcohol misuse is a persistent problem in the UKAF, with estimates forecasting that >50% of those who have left the UKAF meet the criteria for hazardous alcohol use. This is almost double that of the general population [41] and higher in female veterans. Innovative digital interventions can be used to great effect to meet this growing demand for support and treatment for alcohol misuse [42,43].

Currently, there are no smartphone-based alcohol interventions targeting female veterans of the UKAF to reduce alcohol misuse. Therefore, we propose to refine DrinksRation—a theory-driven and user-centered smartphone app—to address this gap for female veterans. Although DrinksRation has been tested in a largely male population, there has been little focus on female veterans. This study protocol describes the design of an RCT to determine the efficacy of DrinksRation among a female veteran population.

The Ministry of Defence Research Ethics Committee and the National Institute for Health and Care Research have identified female veterans as an underserved group regarding support and treatment for alcohol misuse. This is often due to medical services targeting male counterparts, failing to target specific female barriers to care, and a general lack of awareness of female motivations for drinking [17,18,44]. Given the limited evidence to date, there is an urgent need for innovation to support female veterans.

A key strength of this study protocol is the collaboration among the charitable sector, stakeholders, academia, and the UKAF, enabling the development and public release of DrinksRation. A further strength includes remote delivery via a smartphone to overcome issues related to availability or geographic limitations for participants. Furthermore, DrinksRation is unique in that it uses personalized push notifications to promote positive changes in behavior among participants [45]. This ensures longer-term engagement and adherence. DrinksRation can also be used on any iOS and Android device released in the last 7 years. However, owing to personalization, the app requires an active data connection to function.

This study will not be without challenges, the first of which concerns our ability to recruit and retain a sufficient number of female veterans to power our analysis. This is due to a known issue: many of those who have alcohol-related problems are reluctant to seek help to ameliorate their problems. We planned our recruitment approach following best practice and our prior experiences in recruitment to mitigate this issue. Furthermore, the app has been designed to promote its active use, with frequent reminders, which is expected to promote adherence to the app. Second, we expect that some participants may encounter technical issues related to the app (eg, unable to log drinks or receive notifications) or the mobile device. To mitigate potential technical or mobile device issues, we have undertaken extensive testing across a range of popular mobile devices, and we will allow participants to provide in-app feedback that will be regularly monitored by the research team. Third, participants may reduce engagement or disengage in the control arm because BeAlcoholSmart is a static service. We will mitigate this by promoting its use to participants and ensuring that all information and data are presented appropriately. Finally, we acknowledge that our sample comprises participants who may be undergoing active treatment. To mitigate this risk, we will measure and describe the number of participants in each arm already receiving treatment for alcohol.

### Open Access Statement

For the purposes of open access, the author has applied a Creative Commons attribution (CC BY) license to any accepted author manuscript version arising from this submission.

### Conclusions

If this study is successful, the DrinksRation platform along with its refined features could be used not only to support women who have served in the UKAF but also for other conditions and disorders.

## Appendix

Multimedia Appendix 1 Behavior change technique assignment to each DrinksRation component.

Multimedia Appendix 2 Eligibility and consent questionnaire.

Multimedia Appendix 3 Questionnaire set.

Multimedia Appendix 4 Drinks menu alcohol unit assignment.

Multimedia Appendix 5 Peer-review report by the Cabinet Office – Office for Veterans Affairs – UK Government.

## Abbreviations

BCT: behavior change technique
CONSORT: Consolidated Standards of Reporting Trials
CONSORT-EHEALTH: Consolidated Standards of Reporting Trials of Electronic and Mobile Health Applications and Online Telehealth
NHS: National Health Service
PAB: patient advisory board
PMG: project management group
PTSD: posttraumatic stress disorder
PWA: progressive web application
RA: research assistant
RCT: randomized controlled trial
SAB: stakeholder advisory board
TIDieR: Template for Intervention Description and Replication
UKAF: United Kingdom Armed Forces

## Data Availability

Data and code syntax arising from the randomized controlled trial will be made available via the Open Science Framework.

## References

[1] (2019). Population projections: UK armed forces veterans residing in Great Britain, 2016 to 2028. Ministry of Defence.

[2] (2021). We also served: the health and well-being of female veterans in the UK. The Centre for Military Women’s Research.

[3] Chui Z, Leightley D, Jones M, Landau S, McCrone P, Hayes RD, Wessely S, Fear NT, Goodwin L (2023). Mental health problems and admissions to hospital for accidents and injuries in the UK military: A data linkage study. PLoS ONE.

[4] Rhead R, MacManus D, Jones M, Greenberg N, Fear NT, Goodwin L (2020). Mental health disorders and alcohol misuse among UK military veterans and the general population: a comparison study. Psychol Med.

[5] Palmer L, Norton S, Jones M, Rona RJ, Goodwin L, Fear NT (2021). Trajectories of alcohol misuse among the UK Armed Forces over a 12‐year period. Addiction.

[6] Godier-McBard LR, Cable G, Wood AD, Fossey M (2021). Gender differences in barriers to mental healthcare for UK military veterans: a preliminary investigation. BMJ Mil Health.

[7] Irizar P, Leightley D, Stevelink S, Rona R, Jones N, Gouni K, Puddephatt JA, Fear N, Wessely S, Goodwin L (2020). Drinking motivations in UK serving and ex-serving military personnel. Occup Med (Lond).

[8] (2018). Statistics on alcohol, England. NHS Digital.

[9] Murphy D, Spencer-Harper L, Carson C, Palmer E, Hill K, Sorfleet N, Wessely S, Busuttil W (2016). Long-term responses to treatment in UK veterans with military-related PTSD: an observational study. BMJ Open.

[10] Drink less and save money with DrinksRation app. DrinksRation.

[11] Williamson C, Dryden D, Palmer L, Rona R, Simms A, Fear NT, Goodwin L, Murphy D, Leightley D (2022). An expert and veteran user assessment of the usability of an alcohol reduction app for military veterans, Drinks:Ration: a mixed-methods pilot study. Military Behavioral Health.

[12] Leightley D, Williamson C, Rona RJ, Carr E, Shearer J, Davis JP, Simms A, Fear NT, Goodwin L, Murphy D (2022). Evaluating the efficacy of the Drinks:Ration mobile app to reduce alcohol consumption in a help-seeking military veteran population: randomized controlled trial. JMIR Mhealth Uhealth.

[13] Leightley D, Puddephatt J, Jones N, Mahmoodi T, Chui Z, Field M, Drummond C, Rona RJ, Fear NT, Goodwin L (2018). A smartphone app and personalized text messaging framework (InDEx) to monitor and reduce alcohol use in ex-serving personnel: development and feasibility study. JMIR Mhealth Uhealth.

[14] Puddephatt J, Leightley D, Palmer L, Jones N, Mahmoodi T, Drummond C, Rona RJ, Fear NT, Field M, Goodwin L (2019). A qualitative evaluation of the acceptability of a tailored smartphone alcohol intervention for a military population: information about drinking for ex-serving personnel (InDEx) app. JMIR Mhealth Uhealth.

[15] King K, Leightley D, Greenberg N, Fear N (2023). The DrinksRation smartphone app for modifying alcohol use behaviors in UK military service personnel at risk of alcohol-related harm: protocol for a randomized controlled trial. JMIR Res Protoc.

[16] Figueroa CA, Luo T, Aguilera A, Lyles CR (2021). The need for feminist intersectionality in digital health. Lancet Digit Health.

[17] Murphy D, Busuttil W (2014). PTSD, stigma and barriers to help-seeking within the UK Armed Forces. J R Army Med Corps.

[18] Sharp M, Fear NT, Rona RJ, Wessely S, Greenberg N, Jones N, Goodwin L (2015). Stigma as a barrier to seeking health care among military personnel with mental health problems. Epidemiol Rev.

[19] Williamson C, Wickersham A, Sharp M, Dryden D, Simms A, Fear NT, Murphy D, Goodwin L, Leightley D (2023). Loneliness among UK veterans: associations with quality of life, alcohol misuse, and perceptions of partner drinking. J Mil Veteran Fam Health.

[20] Alcohol-use disorders guidance. National Institute for Health and Care Excellence.

[21] Leightley D, Puddephatt J, Goodwin L, Rona R, Fear NT (2018). InDEx: open source iOS and android software for self-reporting and monitoring of alcohol consumption. J Open Res Softw.

[22] Gollwitzer PM, Sheeran P (2006). Implementation intentions and goal achievement: a meta‐analysis of effects and processes. Adv Exp Soc Psychol.

[23] Bell DM, Pahl K (2017). Co-production: towards a utopian approach. Int J Soc Res Methodol.

[24] Sobell LC, Maisto SA, Sobell MB, Cooper A (1979). Reliability of alcohol abusers' self-reports of drinking behavior. Behav Res Ther.

[25] Alcohol guidelines review – report from the guidelines development group to the UK chief medical officers. Department of Health, UK.

[26] Stevelink SA, Jones M, Hull L, Pernet D, MacCrimmon S, Goodwin L, MacManus D, Murphy D, Jones N, Greenberg N, Rona RJ, Fear NT, Wessely S (2018). Mental health outcomes at the end of the British involvement in the Iraq and Afghanistan conflicts: a cohort study. Br J Psychiatry.

[27] Garnett C, Crane D, Michie S, West R, Brown J (2016). Evaluating the effectiveness of a smartphone app to reduce excessive alcohol consumption: protocol for a factorial randomised control trial. BMC Public Health.

[28] Jones N, Whelan C, Harden L, Macfarlane A, Burdett H, Greenberg N (2018). Resilience-based intervention for UK military recruits: a randomised controlled trial. Occup Environ Med.

[29] Williamson C, Rona RJ, Simms A, Fear NT, Goodwin L, Murphy D, Leightley D (2022). Recruiting military veterans into alcohol misuse research: the role of social media and Facebook advertising. Telemed J E Health.

[30] Oetzmann C, White KM, Ivan A, Julie J, Leightley D, Lavelle G, Lamers F, Siddi S, Annas P, Garcia SA, Haro JM, Mohr DC, Penninx BW, Simblett SK, Wykes T, Narayan VA, Hotopf M, Matcham F (2022). Lessons learned from recruiting into a longitudinal remote measurement study in major depressive disorder. NPJ Digit Med.

[31] Arroll B, Goodyear-Smith F, Crengle S, Gunn J, Kerse N, Fishman T, Falloon K, Hatcher S (2010). Validation of PHQ-2 and PHQ-9 to screen for major depression in the primary care population. Ann Fam Med.

[32] Kroenke K, Spitzer RL, Williams JB, Monahan PO, Löwe B (2007). Anxiety disorders in primary care: prevalence, impairment, comorbidity, and detection. Ann Intern Med.

[33] Cloitre M, Shevlin M, Brewin CR, Bisson JI, Roberts NP, Maercker A, Karatzias T, Hyland P (2018). The International Trauma Questionnaire: development of a self-report measure of ICD-11 PTSD and complex PTSD. Acta Psychiatr Scand.

[34] (2010). Readiness ruler. Center for Evidence-Based Practices at Case Western Reserve University.

[35] Saunders Jb, Aasland Og, Babor Tf, De La Fuente Jr, Grant M (1993). Development of the alcohol use disorders identification test (AUDIT): WHO collaborative project on early detection of persons with harmful alcohol consumption--II. Addiction.

[36] EuroQol Group (1990). EuroQol - a new facility for the measurement of health-related quality of life. Health Policy.

[37] Zhou L, Bao J, Setiawan IMA, Saptono A, Parmanto B (2019). The mHealth app usability questionnaire (MAUQ): development and validation study. JMIR Mhealth Uhealth.

[38] Hoffmann TC, Glasziou PP, Boutron I, Milne R, Perera R, Moher D, Altman DG, Barbour V, Macdonald H, Johnston M, Lamb SE, Dixon-Woods M, McCulloch P, Wyatt JC, Chan A, Michie S (2014). Better reporting of interventions: template for intervention description and replication (TIDieR) checklist and guide. BMJ.

[39] Rennie D (2001). CONSORT revised--improving the reporting of randomized trials. JAMA.

[40] Eysenbach G, CONSORT- E (2011). CONSORT-EHEALTH: improving and standardizing evaluation reports of Web-based and mobile health interventions. J Med Internet Res.

[41] McManus S, Bebbington P, Jenkins R, Brugha T (2014). Mental health and wellbeing in England: adult psychiatric morbidity survey 2014. NHS Digital.

[42] Coleman SJ, Stevelink SA, Hatch SL, Denny JA, Greenberg N (2017). Stigma-related barriers and facilitators to help seeking for mental health issues in the armed forces: a systematic review and thematic synthesis of qualitative literature. Psychol Med.

[43] Wickersham A, Petrides PM, Williamson V, Leightley D (2019). Efficacy of mobile application interventions for the treatment of post-traumatic stress disorder: a systematic review. Digit Health.

[44] Sharp M, Serfioti D, Jones M, Burdett H, Pernet D, Hull L, Murphy D, Wessely S, Fear NT (2021). UK veterans’ mental health and well-being before and during the COVID-19 pandemic: a longitudinal cohort study. BMJ Open.

[45] Williamson C, White K, Rona RJ, Simms A, Fear NT, Goodwin L, Murphy D, Leightley D (2022). Smartphone-based alcohol interventions: a systematic review on the role of notifications in changing behaviors toward alcohol. Subst Abus.

