# Supplementary material for: Digital Therapeutic Intervention for Women in the UK Armed Forces Who Consume Alcohol at a Hazardous or Harmful Level: Protocol for a Randomized Controlled Trial
Source: JMIR Res Protoc. 2023 Dec 19;12:e51531. doi: 10.2196/51531 (PMC10762616; doi:10.2196/51531)
Supplement: Multimedia Appendix 2 [file resprot_v12i1e51531_app2.docx]

## Appendix 2: Eligibility and consent questionnaire

Eligibility Questionnaire

1. Do you own an Apple or Android smartphone?
   1. Yes
   2. No
2. Do you live in the UK?
   1. Yes
   2. No
3. What sex were you assigned at birth, on your original birth certificate?
   1. Male
   2. Female
4. How old are you? Please enter an age in the below.
5. Have you ever served in the UK Armed Forces?
   1. Yes
   2. No
   3. Currently serving

Alcohol Screening

Presentation of the Timeline to Follow back for Alcohol.

Consent

1. **Consent 1**: I confirm that I have read and understood the information sheet dated [INSERT DATE AND VERSION NUMBER] for the above project. I have had the opportunity to consider the information and asked questions which have been answered to my satisfaction.
2. **Consent 2**: I consent voluntarily to be a participant in this project and understand that I can refuse to take part and can withdraw from the project at any time, without having to give a reason, up until 1st January 2024
3. **Consent 3**: I consent to the processing of my personal information for the purposes explained to me in the Information Sheet. I understand that such information will be handled under the terms of UK data protection law, including the UK General Data Protection Regulation (UK GDPR) and the Data Protection Act 2018.
4. **Consent 4**: I understand that my information may be subject to review by responsible individuals from King’s College London for monitoring and audit purposes.
5. **Consent 5**: I understand that confidentiality and anonymity will be maintained, and it will not be possible to identify me in any research outputs.
6. **Consent 8**: I agree that the research team may use my data for future research and understand that any such use of identifiable data would be reviewed and approved by a research ethics committee.

If consent provided participants are asked:

1. Please provide your email address so we can send you joining instructions.
