# Supplementary material for: Digital Therapeutic Intervention for Women in the UK Armed Forces Who Consume Alcohol at a Hazardous or Harmful Level: Protocol for a Randomized Controlled Trial
Source: JMIR Res Protoc. 2023 Dec 19;12:e51531. doi: 10.2196/51531 (PMC10762616; doi:10.2196/51531)
Supplement: Multimedia Appendix 3 [file resprot_v12i1e51531_app3.docx]

## Appendix 3: Questionnaire set

Socio-demographics Questionnaire

1. What is your current gender?
   1. 1. Male
   2. 0. Female
   3. -1. Other
2. How old are you?
3. What is your current occupation?
4. Have you served in the Armed Forces?
   1. 1. Yes
   2. 0. No
   3. Yes – Currently serving
5. How long did you serve in the Armed Forces?
6. Which best describes your reason for leaving
   1. 1. Completed term of service
   2. 2. Better employment prospects in civilian life
   3. 3. Impact of Service life on family
   4. 4. Work not exciting or challenging
   5. 5. Dissatisfaction with pay
   6. 6. Lack of promotion prospects
   7. 7. Difficult to plan life outside of work
   8. 8. Due to deployment
   9. 9. Pressure on family
   10. 10. Didn’t want to be aware from home
   11. 11. My service was terminated
   12. 12. Health problems
   13. 13. Pregnancy
   14. 14. Accomplished everything I wanted
7. Are you a reservist?
   1. 1. Yes
   2. 0. No

Depression (PHQ2)

Over the last 2 weeks, how often have you been bothered by the following problems?

| Little interest or pleasure in doing things | Not at all (0) | Several days (1) | More than half the days (2) | Nearly every day (3) |
| --- | --- | --- | --- | --- |
| Feeling down, depressed or hopeless | Not at all (0) | Several days (1) | More than half the days (2) | Nearly every day (3) |

Anxiety (GAD2)

Over the last 2 weeks, how often have you been bothered by the following problems?

| Feeling nervous, anxious or on edge | Not at all (0) | Several days (1) | More than half the days (2) | Nearly every day (3) |
| --- | --- | --- | --- | --- |
| Not being able to stop or control worrying | Not at all (0) | Several days (1) | More than half the days (2) | Nearly every day (3) |

International Trauma Questionnaire for PTSD

Have you been bothered by any of these problem in the past month?

| Having upsetting dreams that replay part of the  experience or are clearly related to the experience? | Not at all (0) | A little bit (1) | Moderately (2) | Quite a bit (3) | Extremely (4) |
| --- | --- | --- | --- | --- | --- |
| Having powerful images or memories that  sometimes come into your mind in which you feel the experience is happening again in the here and now? | Not at all (0) | A little bit (1) | Moderately (2) | Quite a bit (3) | Extremely (4) |
| Avoiding internal reminders of the experience (for example, thoughts, feelings, or physical sensations)? | Not at all (0) | A little bit (1) | Moderately (2) | Quite a bit (3) | Extremely (4) |
| Avoiding external reminders of the experience (for example, people, places, conversations, objects,  activities, or situations)? | Not at all (0) | A little bit (1) | Moderately (2) | Quite a bit (3) | Extremely (4) |
| Being “super-alert”, watchful, or on guard? | Not at all (0) | A little bit (1) | Moderately (2) | Quite a bit (3) | Extremely (4) |
| Feeling jumpy or easily startled? | Not at all (0) | A little bit (1) | Moderately (2) | Quite a bit (3) | Extremely (4) |
| In the past month have the above problems | | | | | |
| Affected your relationships or social life? | Not at all (0) | A little bit (1) | Moderately (2) | Quite a bit (3) | Extremely (4) |
| Affected your work or ability to work? | Not at all (0) | A little bit (1) | Moderately (2) | Quite a bit (3) | Extremely (4) |
| Affected any other important part of your life such as parenting, or school or college work, or other important activities? | Not at all (0) | A little bit (1) | Moderately (2) | Quite a bit (3) | Extremely (4) |

Readiness to Change Ruler

Using the ruler below, indicate how ready you are to make a change to your drinking. If you are not at all ready, you would select 0 and if you are already trying hard to make the change, you would select 10.

Self-efficacy Ruler

Using the ruler shown below, indicate how confident you are about making a change to your drinking. If you are not at all confident about making the change, you would select 0. If you are very confident about making the change, you would select 10.

Alcohol Use Disorder Identification Test

| How often do you have a drink containing alcohol? | (0) Never | (1) Monthly or less | (2) 2 to 4 times a month | (3) 2 to 3 times a week | (4) 4 or more times a week |
| --- | --- | --- | --- | --- | --- |
| How many drinks containing alcohol do you have on a typical day when you are drinking? | (0) 1 or 2 | (1) 3 or 4 | (2) 5 or 6 | (3) 7, 8, or 9 | (4) 10 or more |
| How often do you have six or more drinks on one  occasion? | (0) Never | (1) Less than monthly | (2) Monthly | (3) Weekly | (4) Daily or almost daily |
| How often during the last year have you found  that you were not able to stop drinking once you  had started? | (0) Never | (1) Less than monthly | (2) Monthly | (3) Weekly | (4) Daily or almost daily |
| How often during the last year have you failed to  do what was normally expected from you  because of drinking? | (0) Never | (1) Less than monthly | (2) Monthly | (3) Weekly | (4) Daily or almost daily |
| How often during the last year have you needed  a first drink in the morning to get yourself going after a heavy drinking session? | (0) Never | (1) Less than monthly | (2) Monthly | (3) Weekly | (4) Daily or almost daily |
| How often during the last year have you had a  feeling of guilt or remorse after drinking? | (0) Never | (1) Less than monthly | (2) Monthly | (3) Weekly | (4) Daily or almost daily |
| How often during the last year have you been  unable to remember what happened the night  before because you had been drinking? | (0) Never | (1) Less than monthly | (2) Monthly | (3) Weekly | (4) Daily or almost daily |
| Have you or someone else been injured as a  result of your drinking? | (0) No | (2) Yes, but not in the last year | (4) Yes, during the last year |  |  |
| Has a relative or friend or a doctor or another  health worker been concerned about your drinking or suggested you cut down? | (0) No | (2) Yes, but not in the last year | (4) Yes, during the last year |  |  |

EQ-5D-5L

Under each heading, please tick the ONE box that best describes your health TODAY.

Mobility

1. I have no problems in walking about
2. I have slight problems in walking about
3. I have moderate problems in walking about
4. I have severe problems in walking about
5. I am unable to walk about

Self-Care

1. I have no problems washing or dressing myself
2. I have slight problems washing or dressing myself
3. I have moderate problems washing or dressing myself
4. I have severe problems washing or dressing myself
5. I am unable to wash or dress myself

Usual Activities *(e.g. work, study, housework, family or leisure activities)*

1. I have no problems doing my usual activities
2. I have slight problems doing my usual activities
3. I have moderate problems doing my usual activities
4. I have severe problems doing my usual activities
5. I am unable to do my usual activities

Pain/Discomfort

1. I have no pain or discomfort
2. I have slight pain or discomfort
3. I have moderate pain or discomfort
4. I have severe pain or discomfort
5. I have extreme pain or discomfort

Anxiety/Depression

1. I am not anxious or depressed
2. I am slightly anxious or depressed
3. I am moderately anxious or depressed
4. I am severely anxious or depressed
5. I am extremely anxious or depressed

We would like to know how good or bad your health is TODAY. This scale is numbered from 0 to 100. 100 means the best health you can imagine. 0 means the worst health you can imagine. Please mark an X on the scale to indicate how your health is TODAY. Now, write the number you marked on the scale in the box below.

Risk and Serious Adverse Events Questionnaire (presented as multi choice)

In the last 28 days, have you had:

| Any days off work due to alcohol? | Yes |
| --- | --- |
| Any workdays affected by alcohol? | Yes |
| Any accidents due to alcohol? | Yes |
| Any visits to A&E due to alcohol? | Yes |
| Any police contacts due to alcohol? | Yes |
| Any physical fights due to alcohol? | Yes |
| Any GP visits due to alcohol? | Yes |
| Have you self-harmed as a result of your alcohol use? | Yes |

If any of the above are endorsed. Please provide a brief description.

In the last 28 days, have you experienced an event that:

| Was life threatening (e.g. risk of death or serious impact on life)? | Yes |
| --- | --- |
| Required hospitalization or prolonged an existing hospitalization | Yes |
| Results in persistent or significant disability or incapacity | Yes |

If any of the above are endorsed. Please provide a description of the event including date and time.

mHealth App Usability Questionnaire

| The app was easy to use. | (0) N/A | (1) Disagree) | (2) | (3) | (4) | (5) | (6) | (7) Agree |
| --- | --- | --- | --- | --- | --- | --- | --- | --- |
| It was easy for me to learn to use the app | (0) N/A | (1) Disagree) | (2) | (3) | (4) | (5) | (6) | (7) Agree |
| I like the interface of the app. | (0) N/A | (1) Disagree) | (2) | (3) | (4) | (5) | (6) | (7) Agree |
| The information in the app was well organized,  so I could easily find the information I needed | (0) N/A | (1) Disagree) | (2) | (3) | (4) | (5) | (6) | (7) Agree |
| I feel comfortable using this app in social  settings. | (0) N/A | (1) Disagree) | (2) | (3) | (4) | (5) | (6) | (7) Agree |
| The amount of time involved in using this app  has been fitting for me. | (0) N/A | (1) Disagree) | (2) | (3) | (4) | (5) | (6) | (7) Agree |
| I would use this app again. | (0) N/A | (1) Disagree) | (2) | (3) | (4) | (5) | (6) | (7) Agree |
| Overall, I am satisfied with this app. | (0) N/A | (1) Disagree) | (2) | (3) | (4) | (5) | (6) | (7) Agree |
| Whenever I made a mistake using the app, I  could recover easily and quickly. | (0) N/A | (1) Disagree) | (2) | (3) | (4) | (5) | (6) | (7) Agree |
| This mHealth app provides an acceptable way to  receive healthcare services. | (0) N/A | (1) Disagree) | (2) | (3) | (4) | (5) | (6) | (7) Agree |
| The app adequately acknowledged and provided information to let me know the progress of my action. | (0) N/A | (1) Disagree) | (2) | (3) | (4) | (5) | (6) | (7) Agree |
| The navigation was consistent when moving  between screens. | (0) N/A | (1) Disagree) | (2) | (3) | (4) | (5) | (6) | (7) Agree |
| The interface of the app allowed me to use all  the functions (such as entering information,  responding to reminders, viewing information)  offered by the app. | (0) N/A | (1) Disagree) | (2) | (3) | (4) | (5) | (6) | (7) Agree |
| This app has all the functions and capabilities I  expected it to have. | (0) N/A | (1) Disagree) | (2) | (3) | (4) | (5) | (6) | (7) Agree |
| The app would be useful for my health and wellbeing. | (0) N/A | (1) Disagree) | (2) | (3) | (4) | (5) | (6) | (7) Agree |
| The app improved my access to healthcare  services. | (0) N/A | (1) Disagree) | (2) | (3) | (4) | (5) | (6) | (7) Agree |
| The app helped me manage my health effectively. | (0) N/A | (1) Disagree) | (2) | (3) | (4) | (5) | (6) | (7) Agree |
| The app made it convenient for me to communicate with my healthcare provider | (0) N/A | (1) Disagree) | (2) | (3) | (4) | (5) | (6) | (7) Agree |
| Using the app, I had many more opportunities to  interact with my healthcare provider. | (0) N/A | (1) Disagree) | (2) | (3) | (4) | (5) | (6) | (7) Agree |
| I felt confident that any information I sent to my  provider using the app would be received. | (0) N/A | (1) Disagree) | (2) | (3) | (4) | (5) | (6) | (7) Agree |
| I felt comfortable communicating with my  healthcare provider using the app. | (0) N/A | (1) Disagree) | (2) | (3) | (4) | (5) | (6) | (7) Agree |
