# Supplementary material for: Digital Therapeutic Intervention for Women in the UK Armed Forces Who Consume Alcohol at a Hazardous or Harmful Level: Protocol for a Randomized Controlled Trial
Source: JMIR Res Protoc. 2023 Dec 19;12:e51531. doi: 10.2196/51531 (PMC10762616; doi:10.2196/51531)
Supplement: Multimedia Appendix 4 [file resprot_v12i1e51531_app4.docx]

## Appendix 4: Drinks menu alcohol unit assignment

Based on calculating alcohol unit guidance from the NHS Live Well Service.

| **Type of Drink** | **Number of Unit** |
| --- | --- |
| Single small shot of spirits* (25ml, ABV 40%) | 1 |
| Alcopop (275ml, ABV 5.5%) | 1.5 |
| Small glass of red/white/rosé wine (125ml, ABV 12%) | 1.5 |
| Bottle of lager/beer/cider (330ml, ABV 5%) | 1.7 |
| Can of lager/beer/cider (440ml, ABV 5.5%) | 2 |
| Pint of lower-strength lager/beer/cider (568ml, ABV 3.6%) | 2 |
| Standard glass of red/white/rosé wine (175ml, ABV 12%) | 2.1 |
| Pint of higher-strength lager/beer/cider (568ml, ABV 5.2%) | 3 |
| Large glass of red/white/rosé wine (250ml, ABV 12%) | 3 |

*Gin, rum, vodka, whisky, tequila, sambuca. Large (35ml) single measures of spirits are 1.4 units.
