# Supplementary material for: Digital Therapeutic Intervention for Women in the UK Armed Forces Who Consume Alcohol at a Hazardous or Harmful Level: Protocol for a Randomized Controlled Trial
Source: JMIR Res Protoc. 2023 Dec 19;12:e51531. doi: 10.2196/51531 (PMC10762616; doi:10.2196/51531)
Supplement: Multimedia Appendix 5 [file resprot_v12i1e51531_app5.pdf]

Research Ethics Office

Franklin Wilkins Building
5.9 Waterloo Bridge Wing
Waterloo Road
London SE1 9NH
Telephone 020 7848 4020/4070/4077
rec@kcl.ac.uk

KING'S College LONDON

Daniel Leightley

6 June 2023

Dear Daniel

Reference Number: LRS/DP-22/23-36879

Study Title: Supporting women in the UK Armed Forces who consume alcohol at an increased risk: Refining the DrinksRation platform

Review Outcome: Further amendments/clarifications required before approval can be granted.

Thank you for submitting the above application for ethical approval. Your application has been reviewed and has been approved pending amendments. You are now required to address a number of issues before full approval is granted. These are specified in the feedback table below. Please respond to each point raised by the reviewer and amend your application form, and appendices, accordingly. **Please note that research involving human participants must not commence until your amended application has been reviewed and Full Approval has been granted.**

In order to amend the application, you will simply need to log on onto REMAS and modify the existing application. Once again, your academic supervisor will be required to provide verification.

The submission of your amended application must be accompanied by a cover letter outlining the changes you have made in response to each of the Committee's requests. For ease of completion we recommend that you cut and paste the feedback table from your outcome letter into your cover letter and respond to each point individually. The cover letter should be attached as a Supporting Document in section I9 of your application. Failure to attach a cover letter to your resubmitted application will result in your application being marked as 'Invalid' and returned to you by the Research Ethics Office prior to review.

Please note, once submitted amendments will be reviewed within 15 working days.

If for some reason you choose not to proceed with this research ethics application, please inform the Research Ethics Office.

Yours sincerely,

Ms Jessica Adams

Research Ethics Facilitator

For and on behalf of

PNM Research Ethics Panel

Cc:

Feedback requiring substantial consideration

1. Filter Question 4/Information Sheet. As you identify in the Information Sheet that if you detect participants are drinking at a harmful level you will get in touch with them, please consider whether this may caused stress/anxiety to participants, as this would require high risk review. Please amend your answer to Q4 and select the following option: *D) The study may induce psychological stress or anxiety, or produce humiliation or cause harm or negative consequences beyond the risks encountered in a participants usual everyday life.* A list of committee deadlines and meeting dates can be found here: Submission deadlines and meeting dates | King's College London Intranet. (kcl.ac.uk). If you do not believe that stress/anxiety could be cause, please provide justification outlining why this is the case.

Additional feedback related to application

2. B9. Please clarify when the sign-posting information will be provided to participants.

3.C1. Please clarify how many participants will be invited to interview.

4. C4. It is assumed that you will not be posting adverts on closed social media groups. If you will be recruiting via closed social media groups you will require gatekeeper permission from the group administrator.

5. C10. If a participant withdraws from research it should not be assumed that their data can be retained unless they request otherwise, their data should be withdrawn also unless they give their permission for it to be retained and used.

Research Governance & Data Protection feedback

6. DPIA

- *Retention and deletion, and Privacy notice*:
  - You state that "identifiable data will be retained for 7 years to allow for analyses and scientific publication". - Please check and confirm this is correct as you stated in E9 that identifiable data will not be stored beyond 06/2024. Clarify when all identifiable data will be deleted and how long anonymous data will be archived for.
  - You state that "Online stores will be deleted and offline stored will be deployed using a company with an ISO standard". - Please provide further details as there is no mention of the in the REMAS application. Which company will be used to store offline data? Can you not use KCL platforms to store data?
- *Storage, Access (including third party providers)*
  - You state that "The online database will be stored using Google Firebase servers located within the EU". - Please outline what data will be stored in the online database. What will happen to this data once collected and where it will be stored - KCL OneDrive? How long will this data be stored using google Firebase?

7. PIS

- *What will I be asked to do?* Please state what the " additional data" is in this section which you are consenting to collect via the app.
- *Who will have access to my data and how will it be used?*

  - Please add the updated UK GDPR statement about data processing - "Your data will be processed under the terms of UK data protection law (including the UK General Data Protection Regulation (UK GDPR) and the Data Protection Act 2018)"
  - Please outline where the 3rd party servers are and detail what information they will store and for how long.

- In the PIS for interviews you state "King's College London will keep identifiable information about you for 7 years a er the study has finished." - Please check and confirm this is correct as you stated in E9 that identifiable data will not be stored beyond 06/2024.
- *Will my data be safe?* We recommend clarifying in the PIS how the interviews and focus groups are being transcribed (it is assumed from the application this is by the lead researcher) - If a third-party transcriber service is being used, please update the PIS, and add a statement in the consent form.

Feedback related to recruitment documents

8. Information Sheet - App

- Invitation - Please proof-read and remove duplication's.
- Why have I been invited..?
  - Please expand upon your inclusion/exclusion criteria within this section, it should be clear to potential participants what makes them eligible to participate.
  - Please clarify how many participants will be invited to a follow up interview.
- Please include a separate "Who do I contact for further Information" section and include Dr Leightley's contact details within this section.
- Please include the section "What if something has gone wrong..." section and include the details for the PNM RESC.

"What if I have further questions, or if something goes wrong?

If this project has harmed you in any way or if you wish to make a complaint about the conduct of the project you can contact King's College London using the details below for further advice and information: "

9. Information Sheet - Interviews

- Why have I been invited..? Please ensure it is clear that participants are being invited because they participated in the first stage of the research, using the app.
- What will I be asked to do? Please consider whether you could include some example questions for clarity.
- Please include a separate "Who do I contact for further Information" section and include Dr Leightley's contact details within this section.
- Please include the section "What if something has gone wrong..." section and include the details for the PNM RESC.

"What if I have further questions, or if something goes wrong?

If this project has harmed you in any way or if you wish to make a complaint about the conduct of the project you can contact King's College London using the details below for further advice and information: "

Advice and Comments (do not have to be adhered to, but may help to improve the research)

N/A

Research Ethics
Office

Franklin Wilkins Building
5.9 Waterloo Bridge Wing
Waterloo Road
London SE1 9NH
Telephone 020 7848 4020/4070/4077
rec@kcl.ac.uk

KING'S College LONDON

Daniel Leightley

21/06/2023

Dear Daniel

LRS/DP-22/23-36879: Supporting women in the UK Armed Forces who consume alcohol at an increased risk: Refining the DrinksRation platform

Ethical Clearance

Thank you for submitting your application for the above project. I am pleased to inform you that full approval has been granted by the PNM Research Ethics Panel

Ethical approval has been granted for a period of **5 years** from 21 June 2023. You will not be sent a reminder when your approval has lapsed and if you require an extension you should complete a modification request, details of which can be found here: https://internal.kcl.ac.uk/innovation/governance-ethics-integrity/research-ethics/applications/modifications

Please ensure that you follow the guidelines for good research practice as laid out in UKRIO'sde Code of Practice for research: http://ukrio.org/publications/code-of-practice-for-research/

Any unforeseen ethical problems arising during the course of the project should be reported to the panel Chair, via the Research Ethics Office.

Data Protection Registration

As you have indicated in Section E that personal data will be processed as part of this research project, this letter also confirms that you have also met your requirements for registering this processing activity with King's College London. This is required in line with the College's role as a Data Controller, in accordance with the General Data Protection Regulation (GDPR).

Please note it is the responsibility of the researcher(s) to ensure compliance with other aspects of the GDPR, more information about this can be found here: https://internal.kcl.ac.uk/innovation/governance-ethics-integrity/research-governance-office/data-protection-law-and-research/how-does-uk-dp-law-affect-research

You are required to adhere to all research data/records management and storage procedures agreed to as part of your application. This will be expected even after the completion of the study.

If there are any changes to the project that will impact on how you will you collect, manage or otherwise use your data, these must also be reflected in a modification request as outlined above.

Please note that we may, for the purposes of audit, contact you to ascertain the status of your research.

We wish you every success with your research.

Yours sincerely,

Ms Jessica Adams

Research Ethics Facilitator

For and on behalf of:

PNM Research Ethics Panel
